# CRISPR/Cas9 in cancer therapy: clinical translation, mechanistic strategies, and therapeutic directions

**DOI:** 10.3389/fonc.2026.1888734

**Published:** 2026-07-17

**Authors:** Chu Xin Ng, Sakina Mustafa, Xin Yi Yap, Sau Har Lee

**Affiliations:** 1 School of Biosciences, Faculty of Health and Medical Sciences, Taylor’s University, Selangor, Malaysia; 2 Digital Health and Medical Advancements Impact Lab, Taylor’s University, Selangor, Malaysia; 3 Centre for Active Living, Taylor’s University, Selangor, Malaysia

**Keywords:** cancer therapy, CAR-T cell therapy, clinical trials, CRISPR/Cas9, genome editing, precision oncology

## Abstract

The advent of CRISPR/Cas9 genome editing has significantly transformed the landscape of cancer therapeutics by facilitating precise and programmable manipulation of disease-associated genetic modifications. This review comprehensively evaluates the current clinical and translational landscape of CRISPR/Cas9-based cancer therapies through an analysis of published literature and registered clinical trials. The current CRISPR/Cas9 applications in oncology are primarily centred on three mechanistic strategies: immune cell engineering for enhanced tumor recognition, direct targeting of oncogenic mutations, and modulation of tumor-supportive pathways. Analysis of 32 clinical trials indicates that CRISPR-based interventions have demonstrated encouraging safety profiles and early signs of clinical activity, particularly in *ex vivo* engineered immune-cell therapies. Notable examples include CRISPR-edited CAR-T cell products targeting *CD19* and *BCMA*, which have achieved objective responses in relapsed or refractory hematological malignancies while demonstrating sustained persistence of edited cells *in vivo*. In contrast, clinical translation into solid tumors remains comparatively limited due to challenges associated with delivery efficiency, tumor heterogeneity, and the immunosuppressive tumor microenvironment. Technological advancements, including multiplex genome editing, base editing, and prime editing have expanded the precision and versatility of CRISPR-based interventions, while integration with immunotherapy and nanotechnology-based delivery systems continues to broaden therapeutic potential. Despite these advances, several significant challenges still need to be addressed, including off-target editing, manufacturing scalability, delivery limitations, and regulatory considerations. Overall, CRISPR/Cas9 represents a promising yet evolving platform in oncology, with its future clinical success dependent on achieving a balance between precision, safety, scalability, and long-term therapeutic durability.

## 1 CRISPR/Cas9 as a precision platform in cancer therapy

Cancer remains a major global health burden, characterized not only by its high incidence and mortality but also by its profound biological heterogeneity and adaptive capacity (1). Despite advances in early detection and therapeutic strategies that have improved patient outcomes, conventional treatment modalities such as chemotherapy and radiotherapy continue to be limited by a lack of specificity and associated systemic toxicity (2). These approaches primarily target rapidly proliferating cells, thereby affecting both malignant and normal tissues, and leading to a broad range of adverse effects. More importantly, such treatments do not directly address the underlying genetic and molecular mutations that drive tumor initiation, progression, and therapeutic resistance. This fundamental limitation has driven increasing interest in developing precision-based therapeutic strategies that are capable of selectively modulating disease-relevant molecular pathways and epigenetic regulators (3).

Within this evolving landscape, genome editing technologies have emerged as a promising alternative, with the clustered regularly interspaced short palindromic repeats/CRISPR-associated protein 9 (CRISPR/Cas9) system representing a particularly versatile and widely adopted platform. Derived from the adaptive immune defense mechanisms of bacteria and archaea, CRISPR/Cas9 enables sequence-specific genomic modification through the coordinated action of two fundamental components: the Cas9 endonuclease and a programmable guide RNA (gRNA) (4). The gRNA directs Cas9 to a complementary DNA sequence adjacent to a protospacer adjacent motif (PAM), where the nuclease introduces a targeted double-strand break (DSB). Subsequently, DNA repair is mediated by endogenous cellular mechanisms, including non-homologous end joining (NHEJ) and homology-directed repair (HDR). While NHEJ, which predominates in most cellular contexts, facilitates rapid but error-prone repair that can result in gene-disruptive insertions or deletions, HDR allows for more precise sequence correction through homologous template mediation, although its efficiency is typically restricted by cell cycle constraints (5, 6). The interplay between these pathways highlights the flexibility of CRISPR/Cas9 as both a gene-disruptive and gene-corrective approach.

**Figure 1:**
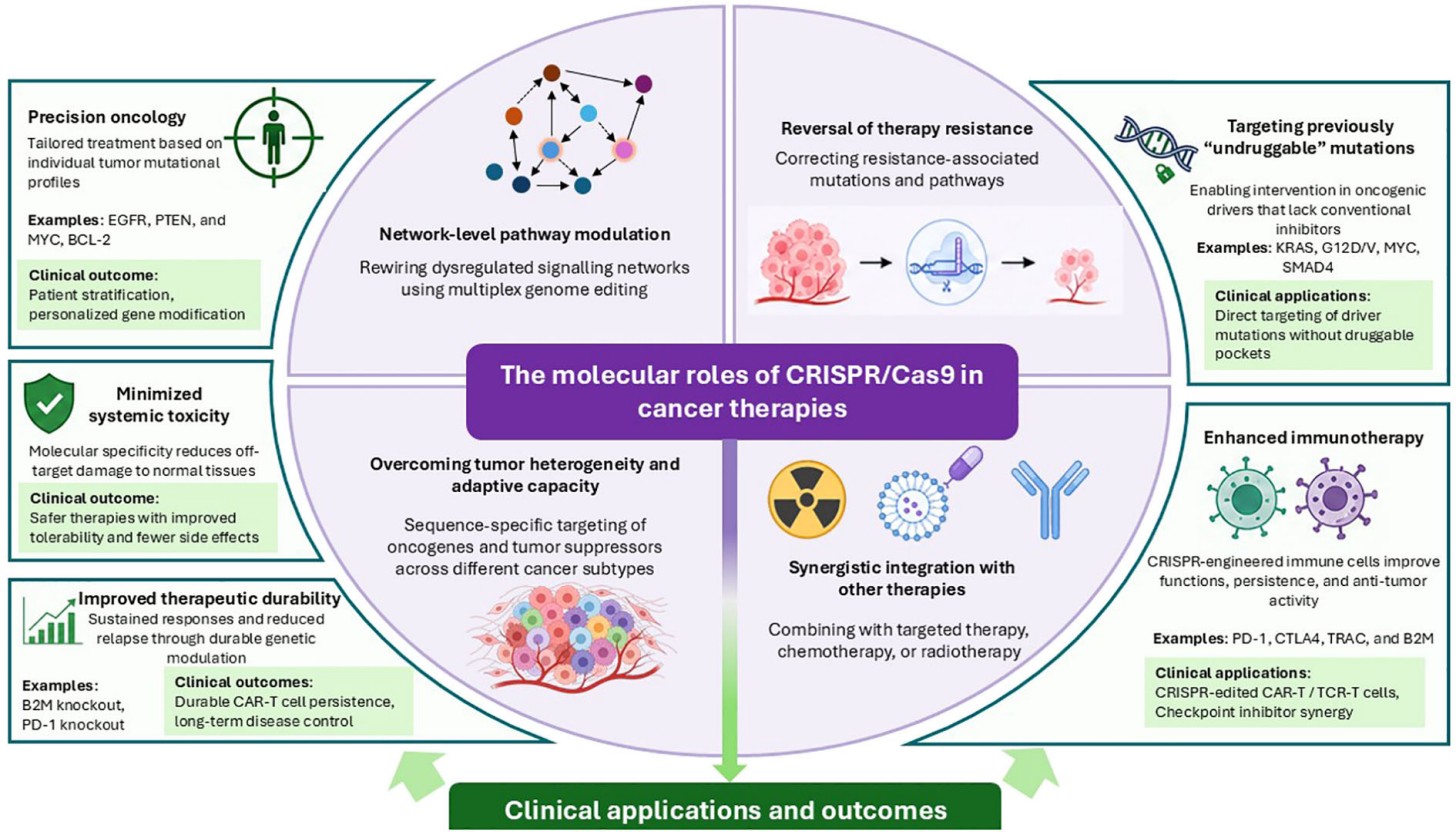
Mechanistic strategies utilizing CRISPR/Cas9 in eradicating cancers. The major mechanistic strategies currently explored in CRISPR/Cas9-based cancer therapies include immune cell modification to enhance tumor recognition and overcome immunosuppressive signaling, direct targeting of oncogenic mutations through both DSB-dependent and -independent editing methods, and modulation of tumor-supportive pathways within the TME.

The growing interest in CRISPR/Cas9 in oncology reflects its advantages to address key limitations of conventional cancer therapies (**Figure 1**). Tumor development is driven by the accumulation of genomic alterations, including activation of oncogenes, loss of tumor suppressor function, and dysregulation of signaling networks that govern proliferation and survival. Unlike chemotherapy and radiotherapy, which exert broad cytotoxic and systemic toxicity effects, CRISPR/Cas9-based approaches offer molecular specificity, enabling direct targeting of oncogenes, tumor suppressors, and regulatory elements implicated in cancer progression (7). In addition, CRISPR allows for programmable and multiplexed genome editing, allowing multiple genetic targets to be modulated simultaneously (8). This is particularly relevant given that tumor progression and therapeutic resistance are rarely governed by single-gene mutations, but instead driven by complex and interconnected signaling networks.

Recent developments have further expanded the role of CRISPR beyond conventional gene disruption toward a broader precision oncology framework that encompasses transcriptional regulation, epigenetic modulation, functional genomic screening, and the development of patient-specific therapeutic strategies. Such versatility is particularly relevant in the context of tumor heterogeneity and adaptive resistance, where simultaneous interrogation and manipulation of multiple molecular pathways may be required to achieve durable therapeutic responses. Consequently, CRISPR is increasingly viewed not only as a therapeutic intervention but also as a platform for target discovery, biomarker identification, and rational therapeutic design in oncology (9).

Importantly, the impact of CRISPR in oncology extends beyond direct therapeutic applications to establishing disease models and target discovery. The generation of genetically defined cellular and animal models using the CRISPR approach has significantly improved the reliability of cancer research, by which tumor biology can be simulated in experimental models. In parallel, high-throughput CRISPR screening approaches have allowed systematic identification of genes involved in tumor progression, immune evasion, and therapeutic resistance, thereby uncovering critical targets that can be exploited for therapeutic development. However, it is worth noting that these advantages of CRISPR/Cas9-based therapies are context-dependent and may remain subject to ongoing clinical validation (10).

Despite these advantages, the clinical translation of CRISPR/Cas9 remains associated with several challenges, including concerns related to off-target editing, delivery efficiency, and long-term genomic stability (11), which will be further discussed in Section 4. While technological innovations such as high-fidelity Cas variants and alternative editing platforms have begun to address these limitations, the safe and effective application of CRISPR in oncology requires continued optimization within complex biological systems (12). As such, CRISPR should be viewed not simply as a standalone gene-editing tool but as a multifaceted and evolving therapeutic platform that can be potentially integrated within the broader framework of cancer treatment.

### 1.1 Literature search strategy and study selection

This review was conducted through a structured literature search to identify published studies and registered clinical trials evaluating the application of CRISPR/Cas9 in cancer research and therapy. Authoritative databases, including PubMed and Web of Science, were searched for relevant articles published between 2013 and 2026, corresponding to the period following the introduction of CRISPR/Cas9 as a genome editing tool. Search terms were developed using combinations of keywords and Medical Subject Headings (MeSH) related to CRISPR and cancers, including “CRISPR/Cas9”. “Genome editing”, “cancer therapy”, “oncology”, “CAR-T”, “immunotherapy”, “tumor microenvironment”, “clinical trial”, “hematological malignancies”, “solid tumors”, and “precision oncology”. Boolean operators (“AND”, “OR”) were applied to refine search results.

Registered interventional studies were identified through ClinicalTrials.gov, supplemented by information from published trial reports, conference proceedings, and regulatory updates when available. Clinical trials investigating CRISPR-based therapeutic interventions in cancers were included regardless of recruitment status, including completed, recruiting, active, terminated, withdrawn, or not yet recruiting. Studies were included if they: (i) investigated CRISPR/Cas9 or related CRISPR-based editing technologies in cancer research or therapy; (ii) reported mechanistic, translational, preclinical, or clinical findings relevant to oncology; or (iii) described ongoing or completed oncology-related clinical trials. Studies focusing exclusively on non-oncological diseases, non-CRISPR gene-editing platforms, or lacking sufficient methodological information were excluded. Non-English publications, editorials, commentaries, and duplicate records were also excluded where appropriate.

The retrieved literature was screened for relevance through title, abstract, and full-text evaluation. Priority was given to peer-reviewed original research articles, clinical trial publications, high-impact reviews, and recent studies that provided insights into therapeutic mechanisms, clinical translation, safety considerations, and emerging developments in CRISPR/Cas9-based cancer therapies. The selected studies were subsequently synthesized narratively to provide a comprehensive overview of the current state and future directions of CRISPR/Cas9 in cancer therapeutics.

## 2 Clinical translation of CRISPR/Cas9 in oncology

### 2.1 Clinical trajectory of CRISPR/Cas9 in cancer treatment

The clinical implementation of CRISPR/Cas9 in oncology represents an important convergence of molecular precision and real-world medical translational goals. Since the first-in-human trial (NCT02793856) demonstrated the feasibility in Programmed Cell Death-1 (*PDCD1*)-Disrupted T cells (13), researchers have progressively developed a range of clinical strategies to harness CRISPR for different types of cancer. As of 2025, over a dozen registered interventional trials (**Table 1**) are exploring CRISPR-based interventions in various cancer types, from early-phase dose-escalation studies to adaptive trials incorporating multi-gene editing techniques.

**Table 1:** CRISPR/Cas9-based cancer therapies in clinical trials.

Trial ID	Year	Phase	Status	Intervention name	Cancer type	CRISPR strategy	Key target(s)
NCT02793856	2016	I	Completed	–	NSCLC	*Ex vivo* gene knock out	*PDCD1*
NCT03081715	2017	I	Completed	–	Esophageal cancer	*Ex vivo* gene knock out	*PDCD1*
NCT02863913	2016	I	Withdrawn	–	Bladder cancer	*Ex vivo* gene knock out	*PDCD1*
NCT06783270	2024	I	Recruiting	–	Melanoma	PD-1 edited TIL therapy	*PDCD1*
NCT03044743	2017	I/II	Unknown	–	EBV-associated cancers	Autologous PD-1 EBV-specific CTL editing	*PDCD1*
NCT02867332	2016	I	Withdrawn	–	Renal cell carcinoma	Autologous PD-1 knock out T-cells.	*PDCD1*
NCT04417764	2019	I	Recruiting	–	Advanced hepatocellular carcinoma	*Ex vivo* PD-1 knock out	*PDCD1*
NCT03545815	2018	I	Unknown	MPTK-CAR-T cells	Mesothelin positive solid tumors.	Mesothelin-specific CAR-T	*TRAC, PDCD1*
NCT03747965	2018	I	Unknown	GC008t	Mesothelin positive solid tumors	PD-1 knock out	*PDCD1*
NCT05812326	2019	I/II	Completed	AJMUC1	Breast cancer	Anti-MUC1 CAR-T with PD-1 knock out	*PDCD1, MUC1*
NCT03399448	2018	I	Terminated	HLA-A*0201 restricted NY-ESO-1 redirected T cells	Multiple myeloma and sarcoma	TCR editing	*PDCD1, TCRα, TCRβ*
NCT04637763	2021	I	Ongoing	CB-010	Relapsed or refractory B Cell Non-Hodgkin Lymphoma	Allogeneic CAR-T therapies	*CD19, TRAC, PD-1*
NCT05722418	2023	I	Recruiting	CB-011	Relapsed or refractory multiple myeloma	Allogeneic anti-BCMA CAR-T therapy	B-cell maturation antigen (BCMA), *TRAC, B2M*
NCT06128044	2024	I	Terminated	CB-012	Relapsed or refractory acute myeloid leukemia	Allogeneic CAR-T therapy	C-type lectin-like molecule-1 (*CLL-1*), *PDCD1*, *B2M*
NCT03398967	2018	I/II	Completed	CTA101	Relapsed or refractory Leukemia or Lymphoma	Allogeneic dual-targeted CAR-T therapies	*CD19* and *CD20*, or *CD22*, *CD52*, *TRAC*
NCT04035434	2019	I/II	Terminated	CTX110	Relapsed or refractory B-cell malignancies	Allogeneic CAR-T therapies	*CD19*, *B2M*, *TRAC*
NCT05643742	2023	I/II	Recruiting	CTX112	Relapsed or refractory B cell malignancies	Allogeneic CAR-T therapies	*CD19*, *TRAC*, *B2M*, *TGFBR2*, Regnase-1
NCT04502446	2020	I	Terminated	CTX130	Relapsed or refractory T or B Cell Malignancies	Allogeneic CAR-T therapy	*CD70*, *TRAC, B2M*
NCT06492304	2024	I/II	Completed	CTX131	Relapsed/refractory hematological malignancies	Allogeneic CAR-T therapies	*CD70*, *TRAC, B2M*
NCT05037669	2022	I	Withdrawn	PACE CART19	LCD19+ leukemia and lymphoma	Allogeneic CAR-T therapy	*CD19*, *B2M, CIITA*, and *TRAC*.
NCT03166878	2017	I/II	unknown	UCART019	B-cell malignancies	CAR-T editing	*CD19*, *TRAC, B2M*
NCT04037566	2019	I	Recruiting	XYF19 CAR-T cells	CD19+ leukemia or lymphoma	Autologous CAR-T therapy	*CD19*, hematopoietic progenitor kinase 1 (*HPK1*)
NCT04557436	2020	I	Completed	TT52CAR19	B-cell Acute Lymphoblastic Leukemia	Allogeneic anti-CD19 CAR-T therapy	*CD19, TRAC, CD52*
NCT05662904	2028	I	Not yet recruiting	–	Acute myeloid leukemia	Allogeneic CD33 knocked out in CD34+hematopoietic stem cells (HSC)	*CD33*
NCT05566223	2023	I/II	Withdrawn	–	NSCLC	CISH-edited TIL therapy	*CISH*
NCT04426669	2020	I/II	Completed	–	Gastrointestinal cancers	CISH-edited TIL therapy	*CISH*
NCT06726564	2024	I	Recruiting	MT 027	Solid tumors	Allogeneic CAR-T therapy	*B7H3*
NCT06742593	2025	I	Not yet recruiting	MT 027	Brain, meninges, and spinal cord metastatic solid tumors.	Allogeneic CAR-T therapy	*B7H3*
NCT05066165	2021	I/II	Terminated	NTLA-5001	Acute myeloid leukemia	Autologous TCR therapy	Wilms’ tumor 1 (*WT1*)
NCT06846424	2025	I	Not yet recruiting	SCT-001	Ovarian cancer, fallopian tube cancer, and peritoneal cancer	CAR-T editing	Tumor-associated glycoprotein 72 (TAG-72)
NCT06815029	2026	I	Recruiting	–	Glioblastoma, grade 3 or 4 IDH-mutant astrocytoma	Autologous TGFβR2-knock out with IL13Rα2-CAR T cells	*IL13Rα2*
NCT03057912	2018	I	Unknown	TALEN	HPV-associated cervical cancers	Direct gene targeting	HPV *E6/E7*

Most current efforts (such as NCT04037566, NCT04426669, NCT04417764) have focused on *ex vivo* genome editing, whereby autologous or allogeneic T cells are harvested, genetically modified using CRISPR/Cas9, and reinfused into patients via various approaches, including intrathecal or intracerebroventricular approaches (14–16). This approach circumvents many of the systemic delivery challenges associated with *in vivo* editing, while enabling better control over editing efficiency and cell expansion. Early trials, for example, those targeting *PDCD1* in non-small cell lung cancer (NSCLC) and esophageal squamous cell carcinoma, mainly focused on safety and feasibility endpoints (13). Interestingly, these studies established foundational parameters, including acceptable tolerability of CRISPR-modified cells, absence of severe cytokine release syndrome (CRS), and preliminary evidence of anti-tumor activity (16, 17). Building on that, later studies (NCT05037669, NCT06128044, NCT05722418, NCT03166878, NCT06492304, NCT05643742, and NCT04035434) have adopted increasingly complex editing strategies, incorporating multiplex editing to target multiple genes at once. This includes targeting of *PDCD1*, TCRαβ (*TRAC*), and Beta-2 microglobulin (*B2M*) to simultaneously remove immune checkpoints, T cell receptors, and MHC class I expression. Such modifications have enabled the generation of “off-the-shelf” universal chimeric antigen receptor (CAR)-T cells that avoid graft-versus-host disease (GvHD) and recipient immune rejection (18).

Although most CRISPR-based trials are still in the early stages (Phase I or I/II), several have progressed toward impactful outcomes, including persistence of edited T cells, early evidence of antigen-specific tumor clearance, and modest improvement in progression-free survival (18, 19). This transition reflects increasing confidence in CRISPR’s clinical safety profile, supported by stringent regulatory standards and the latest off-target surveillance, such as the use of GUIDE-seq and CIRCLE-seq (20, 21), alongside high-fidelity Cas9 variants and improved guide RNA design, which have significantly enhanced editing specificity while minimizing unintended genomic alterations (22).

One particular area that is gaining traction in the clinical trajectory has been the adoption of CRISPR for viral oncogene targeting, particularly in human papillomavirus (HPV)-driven cervical and anal cancers. For example, the trial NCT03057912 is pioneering the use of CRISPR to silence HPV *E6*/*E7* genes, which are the key oncogenic drivers that are refractory to classical therapies (23). This broadens the potential uses of CRISPR beyond immunotherapy, moving into the direct oncogene silencing application. Despite its promise, CRISPR/Cas9 therapy in cancer is not without limitations. As discussed in our subsequent section (Section 4), clinical scalability for CRISPR remains challenged by manufacturing costs, editing consistency, and quality control bottlenecks. Moreover, the long-term consequences of genomic disruption, especially in non-coding regulatory regions or enhancers, are not yet fully understood (24). While short-term toxicity profiles are encouraging, ongoing trials must integrate rigorous long-term follow-up, including insertional mutagenesis screening and secondary malignancy risk monitoring (24). In addition, it is also noteworthy that *in vivo* CRISPR applications for cancer have yet to gain clinical recognition, primarily due to the lack of delivery vectors with tumor-specific targeting sites and immunological neutrality. Nonetheless, several preclinical advances such as tumor-targeted or stimuli-responsive nanoparticles, engineered exosomes, and base editing, could potentially redefine the horizon of CRISPR-enabled oncologic interventions (25, 26).

Although the current clinical progress of CRISPR/Cas9-based cancer therapies remains dominated by early-phase studies, several important translational insights can already be identified from the studies summarized in **Table 1**. A notable observation is that the majority of studies have prioritized safety, feasibility, manufacturing consistency, and persistence of edited cells as their primary endpoints (13, 27), whereas robust efficacy endpoints such as objective response rate (ORR), progression-free survival (PFS), and overall survival (OS) remain sparsely reported. This reflects the developmental stage of the field, where establishing the safety of genome editing in humans remains a prerequisite for broader clinical implementation (24).

One of the clearest trends emerging from completed and ongoing studies is the differential clinical performance between hematological malignancies and solid tumors. Trials involving B-cell malignancies, multiple myeloma, acute lymphoblastic leukemia, and lymphoma have generally demonstrated more encouraging outcomes than those conducted in solid tumors. This disparity is likely attributable to biological rather than technological factors. Hematological cancers provide readily accessible tumor targets, exhibit relatively homogeneous antigen expression, and benefit from established adoptive cell therapy infrastructure (28). Consequently, CRISPR editing can be integrated into clinically validated CAR-T platforms targeting *CD19, BCMA, CD70*, or *CD33* while maintaining efficient *ex vivo* quality control and expansion prior to reinfusion. For example, the CTX110, CTX112, CB-010, CB-011, and UCART019 programs collectively demonstrate the transition of CRISPR from proof-of-concept immune editing toward scalable allogeneic cellular therapies through simultaneous disruption of *TRAC* and *B2M* to minimize GvHD and host immune rejection (**Table 1**). These studies highlight that the most successful applications of CRISPR to date are those that enhance an already effective immunotherapeutic platform rather than relying solely on genome editing as an independent intervention.

In contrast, clinical outcomes in solid tumors have been considerably more variable. Early studies targeting *PDCD1* in NSCLC (NCT02793856) and esophageal cancer (NCT03081715) successfully demonstrated the feasibility and acceptable safety of CRISPR-edited T-cell infusion, with no evidence of severe genome editing-related toxicities during the observation period (19). However, therapeutic responses remained modest despite successful cell manufacturing and infusion. Similar observations were reported in mesothelin-targeting CAR-T studies, where acceptable safety profiles were accompanied by limited tumor regression and relatively low persistence of engineered cells. For example, the MPTK-CAR-T trial (NCT03545815) reported favourable tolerability with no significant cytokine release syndrome (CRS) or neurotoxicity but demonstrated minimal objective tumor regression, whereas the subsequent GC008T study (NCT03747965) achieved disease stabilization in several patients but limited evidence of durable tumor eradication (**Table 1**). These findings suggest that successful genome editing alone is insufficient to overcome barriers imposed by tumor heterogeneity, poor immune-cell trafficking, stromal exclusion, metabolic constraints, and immunosuppressive signaling within the tumor microenvironment (TME) (19, 28).

Safety findings across trials have generally been encouraging and represent one of the strongest translational achievements of the field. Completed studies involving *PDCD1*-disrupted T cells established the feasibility of *ex vivo* CRISPR editing with manageable toxicity profiles and no unexpected severe adverse events directly attributable to genome editing. Similarly, more recent allogeneic CAR-T programs have not identified major safety signals associated with multiplex editing strategies involving simultaneous disruption of immune checkpoints, endogenous T-cell receptors, and major histocompatibility pathways (29, 30). Nevertheless, interpretation of these findings should be interpreted cautiously because long-term surveillance data remain limited. Most studies have relatively short follow-up durations, and the potential consequences of chromosomal rearrangements, clonal expansion, insertional events, or delayed genomic instability remain incompletely characterized.

Another important lesson emerging from the current clinical trials concerns the persistence of edited cells. Durable persistence has consistently correlated with improved therapeutic activity, particularly in hematological malignancies (15, 18). Accordingly, many next-generation programs have shifted beyond simple checkpoint disruption toward multiplex editing strategies that simultaneously enhance persistence, reduce exhaustion, and improve resistance to tumor-mediated immunosuppression. The incorporation of additional targets such as *TGFBR2*, Regnase-1, *HPK1*, and *CISH* in newer studies reflects a growing recognition that sustained anti-tumor efficacy requires optimization of multiple biological processes rather than correction of a single inhibitory pathway.

Importantly, trial termination should not automatically be interpreted as clinical failure. Several withdrawn or terminated studies within the CRISPR-related clinical trials appear to reflect evolving development strategies rather than evidence of unacceptable safety or inefficacy. Some trials may be withdrawn or terminated due to sponsor reprioritization, overlapping product pipelines, slow recruitment, manufacturing challenges, regulatory redesign, or strategic transition toward next-generation edited products (31). This is especially apparent among allogeneic CAR-T platforms, where companies often discontinue earlier products after developing more refined constructs with improved editing design, antigen selection, or safety features. For example, CRISPR Therapeutics announced the strategic transition from first-generation allogeneic CAR-T products, CTX110 and CTX130, to the next-generation candidates CTX112 and CTX131, which were engineered to enhance anti-tumor potency, improve cellular persistence, and reduce T-cell exhaustion (32). This iterative pattern highlights the rapid evolution of the field, where clinical development frequently progresses through platform optimization rather than direct linear advancement of individual products.

Future studies should therefore standardize reporting of editing efficiency, objective response rates, persistence kinetics, off-target surveillance, and long-term follow-up, which will be essential for determining the true therapeutic contribution of CRISPR-mediated genome editing in cancer treatment.

### 2.2 Therapeutic targeting across cancer types

To date, the current clinical landscape of CRISPR/Cas9-based cancer therapies reflects a strong focus on hematological malignancies, whereas the use in solid tumors is steadily growing. This distribution is not coincidental, but rather reflects differences in biological accessibility, microenvironmental complexity, and feasibility of gene-editing delivery strategies. As summarized in **Table 1**, the therapeutic targeting of CRISPR interventions can be broadly grouped into hematologic cancers, solid tumors, and virus-associated malignancies, each presenting distinct opportunities and limitations.

Hematological malignancies, including acute lymphoblastic leukemia (ALL), non-Hodgkin lymphoma, and multiple myeloma, represent the most advanced and clinically relevant targets for CRISPR-based treatment studies (15, 33). For instance, NCT04035434, NCT05037669, NCT04557436, NCT03399448, and NCT05722418. The underlying reasons for the advancement in these CRISPR-based treatment studies are mainly due to the fact that these hematological cancers are generally characterized by the relative accessibility of malignant cells in the blood circulatory system, and the well-established infrastructure for *ex vivo* manipulation of immune cells, particularly T lymphocytes. Consequently, many trials have focused on engineering CAR-T or T-cell receptor (TCR)-modified T cells using CRISPR to enhance immune cells function and persistence (8, 29). In addition, early-phase clinical data also indicate that such approaches may improve anti-tumor responses, while supporting the development of standardized allogeneic genetic-editing techniques with broader clinical applicability (27).

In contrast, the application of CRISPR/Cas9 in solid tumors, including lung cancer, breast cancer, pancreatic cancer, and glioblastoma, remains comparatively challenging and is still largely exploratory. Unlike hematological cancers, solid tumors are characterized by heterogeneous cellular composition, dense extracellular matrices, and immunosuppressive TMEs, all of which hinder efficient delivery and functional activity of gene-editing systems (28). Nevertheless, several trials have attempted to overcome these barriers by employing CRISPR-edited immune cells targeting tumor-associated antigens such as MUC1 (NCT05812326) and mesothelin (NCT03545815), or by knocking out inhibitory pathways to enhance T cell persistence and cytotoxicity (34). While the therapeutic efficacy has remained modest, this highlight the need for continued optimization of delivery systems, tumor penetration strategies, and combinatorial approaches in order to overcome immunosuppressive barrier (34).

Furthermore, a particularly promising niche within CRISPR-based cancer treatment is the targeting of virus-associated cancers, especially those driven by HPV and, in rare cases, Epstein-Barr virus (EBV). In HPV-associated cancers, including cervical, anal, and certain head and neck carcinomas, the viral proteins E6 and E7 play a central role in tumorigenesis by inactivating key tumor suppressors such as p53 and retinoblastoma protein (Rb). Therefore, this makes them ideal candidates for direct gene disruption. Clinical investigations, such as NCT03057912, have demonstrated the feasibility of using CRISPR to selectively inactivate these viral drivers, which represents a transition from indirect immunomodulation to direct genomic inactivation of viral-related oncogenic drivers (35). Moreover, viral-oncogenic drivers possess highly selective targets since they are foreign to the human genome, thereby minimizing concerns of off-target editing within essential host genes.

In the context of EBV-associated malignancies, including nasopharyngeal carcinoma and certain lymphomas, therapeutic development has primarily been driven by adoptive immunotherapy approaches (36). For example, clinical trial NCT03044743 utilizes CRISPR-mediated PD-1 knockout EBV-specific cytotoxic T lymphocytes (EBV-CTLs) to selectively target tumor cells expressing viral antigens (37). While this strategy demonstrates the clinical feasibility of exploiting viral antigens for tumor targeting, it remains dependent on effective antigen presentation and may be constrained by tumor immune evasion. In contrast, preclinical studies have demonstrated that targeting EBV latent genes (such as *EBNA1*, *LMP1*, and *LMP2*) can lead to the loss of viral episomes within host cells, thereby inhibiting tumor cell proliferation and promoting apoptosis (38, 39). This approach shows potential for a shift in treating EBV-related cancers, from immune-mediated clearance to elimination of non-host oncogenic driver, thereby potentially overcoming limitations associated with CTL-based CRISPR/Cas9 treatment.

Overall, the development of CRISPR/Cas9 interventions across the cancer therapeutic area reflects a field that is both strategically focused and increasingly diversifying. CRISPR/Cas9 treatment in hematological malignancies may continue to lead due to their compatibility with *ex vivo* editing platforms and immunotherapeutic integration. Meanwhile, the CRISPR/Cas9 approach focusing on solid tumors may advance substantially with continued technological innovation, particularly in delivery and tumor penetration. Additionally, virus-associated cancers highlight a unique opportunity for precision targeting of non-host oncogenic elements (35).

### 2.3 Methodologies, frameworks, and target selection strategies

Building upon the clinical landscape outlined in Section 2.1, current CRISPR/Cas9-based cancer treatment studies reflect a convergence toward specific methodological and targeting strategies. Rather than functioning solely as a genome-editing tool, CRISPR is increasingly being utilized as a programmable multiplex engineering platform, whereby multiple genetic loci are simultaneously modified to achieve synergistic functional effects (30, 40).

A key methodological highlight from these studies is the predominance of *ex vivo* genome editing, which prioritizes the ease of manipulation, safety, and reproducibility (41). As highlighted in Section 2.1, this approach enables precise manipulation of immune cells before reinfusion, thereby reducing systemic exposure and allowing rigorous validation of editing outcomes. This transition underscores a broader recognition that effective cancer therapy requires coordinated modulation of interconnected pathways, rather than isolated genetic interventions (41). Consequently, CRISPR-based methodologies are increasingly designed to integrate multiple layers of functional enhancement within a single therapeutic construct.

At the level of target selection, current CRISPR-based oncology strategies are focusing on immune-centric strategies and determinants of cellular compatibility (40). The most prominent among these are immune checkpoint regulators, including *PDCD1, CTLA-4, LAG-3*, and *TIM-3*, which play central roles in mediating T cell exhaustion and tumor immune evasion In parallel, intracellular modulators such as CISH are increasingly explored to further enhance cytokine signaling and T cell persistence (**Table 1**). Importantly, these modifications reflect a strong emphasis on the importance of multiplex genetic engineering to restore functional immune competence within tumor environments.

In addition to immune activation, another major focus involves improving the scalability and compatibility of engineered cellular therapies. Targets such as *TRAC* and *B2M* are frequently incorporated into multiplex editing framework to reduce the risk of GvHD and host immune rejection (40, 42). Importantly, these modifications are rarely implemented individually, but instead integrated alongside checkpoint disruption and other functional enhancements within increasingly complicated editing strategies.

Overall, these highlight that CRISPR/Cas9 does not function solely as a precision editing tool in cancer treatment, but rather as an integrative platform for complex genetic modifications to reprogramme cellular behavior.

## 3 Mechanistic basis of CRISPR/Cas9 therapeutic strategies

**Figure 2:**
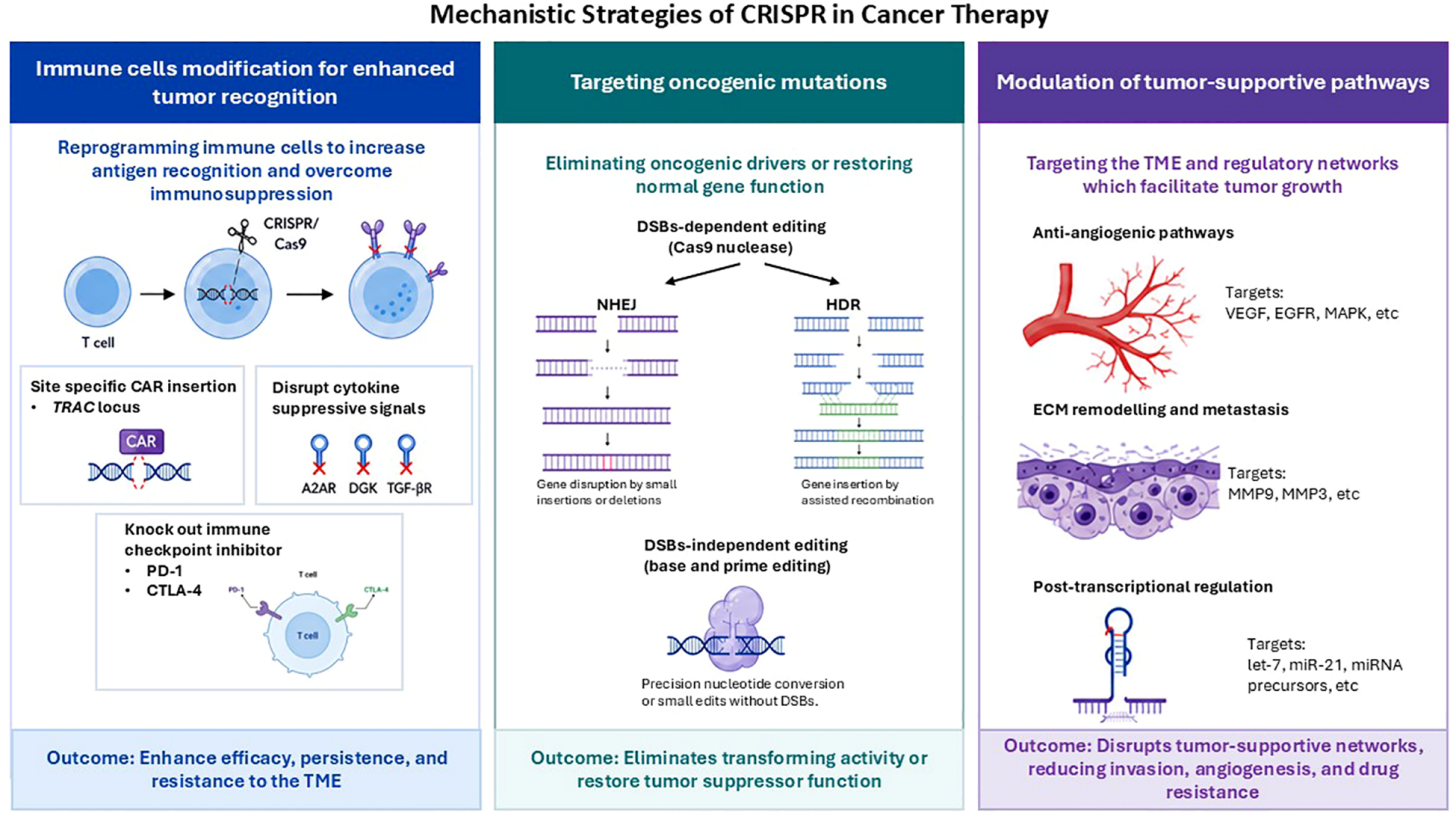
Mechanistic and translational advantages of CRISPR/Cas9 in cancer therapy. This figure summarizes the major advantages of CRISPR/Cas9-based cancer therapies across molecular, cellular, and clinical levels. CRISPR/Cas9 enables precision oncology through sequence-specific targeting of oncogenic alterations, while reducing systemic toxicity associated with conventional therapies. The platform also supports multiplex genome editing for network-level pathway modulation, reversal of therapy resistance, and targeting of previously “undruggable” mutations. In addition, CRISPR-based approaches can improve therapeutic durability and enhance anti-tumor immunity through engineered immune cell strategies.

The clinical application of CRISPR/Cas9 in oncology is characterized by the integration of mechanistically distinct yet functionally convergent strategies that target different levels of tumor biology. Generally, these approaches can be categorized into immune cell reprogramming for enhanced tumor recognition, direct correction or disruption of oncogenic mutations, and targeting tumor-supportive pathways within the TME (**Figure 2**). Although conceptually discrete, these strategies are often applied in combination, reflecting the need for coordinated intervention across multiple biological processes involved in tumor growth and progression. Notably, immune cell editing has emerged as the most clinically advanced modality, largely due to the feasibility of *ex vivo* manipulation and the ability to circumvent delivery constraints associated with *in vivo* editing (40, 41). In contrast, approaches involving direct tumor editing remain technically challenging, particularly due to limitations in achieving efficient and tumor-specific delivery, as well as uncertainty in DNA repair outcomes following editing (41). Across all approaches, concerns related to off-target editing and genomic instability remain significant, necessitating the importance of continued optimization of editing platforms and the development of robust validation and monitoring strategies.

### 3.1 Editing immune cells for enhanced tumor recognition

Among the various CRISPR-based therapeutic strategies, the genetic reprogramming of immune cells represents the most clinically advanced approach. This strategy is fundamentally rooted in improving tumor antigen recognition, enhancing immune persistence, and reducing resistance to immunosuppressive signaling, thereby addressing key limitations of conventional adoptive cell therapies. A key feature of this strategy is the site-specific integration of CARs, most commonly within the *TRAC* locus (43). The molecular mechanism involves gRNA-directed Cas9 cleavage within the *TRAC* locus, followed by NHEJ-mediated repair that introduces a frameshift mutation, preventing functional TCR assembly and surface expression. This targeted insertion not only ensures uniform CAR expression but also disrupts endogenous T cell receptor signaling (43, 44). Importantly, this approach supports more uniform receptor expression and reduces risk associated with random integration and TCR mispairing, reflecting a shift from random transgene integration toward genome-guided engineering (44).

In parallel, the disruption of immune checkpoint pathways, particularly *PDCD1* and *CTLA-4*, has been consistently applied to restore T cell effector function (45, 46). Rather than acting through simple gene deletion, these modifications effectively restore intracellular signaling networks by removing inhibitory feedback loops that constrain activation, proliferation, and cytokine production. Mechanistically, this results in sustained activation of downstream pathways, including enhanced interferon-γ secretion and improved cytotoxic responses, thereby overcoming tumor-induced immune exhaustion (45, 46).

Beyond checkpoint modulation, emerging strategies increasingly incorporate multiplex genome editing to remodel the TME response. Targets such as Adenosine A2A Receptor (*A2AR*), Diacylglycerol Kinase (*DGK*), and components of the Transforming Growth Factor Beta (TGF-β) signaling axis exemplify efforts to neutralize extrinsic suppressive factors within the TME (47–49). Specifically, elevated levels of adenosine are often observed within tumors, which bind to *A2AR* and suppress T cell activation and cytokine production within the TME (48). Similarly, *DGK* is an enzyme that phosphorylates diacylglycerol to phosphatidic acid, and its activation downregulates distal signaling molecules of the TCR, resulting in diminished T cell responses (49). Additionally, TGF-β functions as an inhibitory cytokine in the TME, promoting T cell exhaustion and development of regulatory T cells, thereby contributing to immune evasion by tumors (47). Utilizing CRISPR/Cas9 for multiplexed gene editing, a direct Cas9 cleavage to both the TCR alpha (*TRAC*) and TCR beta (*TRBC*) constant region genes can be done, thereby preventing endogenous TCR expression and eliminating the risk of TCR mispairing (50). These approaches reflect a growing recognition that effective immunotherapy requires not only enhanced antigen recognition but also resilience against inhibitory metabolic and cytokine-mediated signals.

### 3.2 Targeting oncogenic modifications through genome editing

In contrast to immune-based strategies, the direct targeting of oncogenic mutations represents a more technically demanding application of CRISPR/Cas9, aimed at eliminating oncogenic drivers or restoring normal gene function. Traditional CRISPR/Cas9 editing relies on the induction of DNA DSBs, which are repaired primarily through either NHEJ or HDR (51). While NHEJ enables efficient gene disruption through the introduction of frameshift mutations, its error-prone nature limits its utility for precise gene correction (52). Conversely, HDR allows accurate repair using a homologous template but is restricted by cell cycle dependence and inherently low efficiency (53). This contrast has constrained the clinical applicability of DSB-dependent editing for precise oncogenic correction.

To address these limitations, increasingly attention has shifted toward DSB-independent editing platforms, including base editing and prime editing. Base editors employ a catalytically inactive Cas9 (dCas9) conjugated with cytidine deaminase enzymes to enable precise nucleotide conversions, such as cytosine (C) to thymine (T) or adenine (A) to guanine (G) substitutions, without inducing DSBs (54). Conversely, prime editing utilizes a more advanced molecular mechanism, incorporating a Cas9 nickase fused to a reverse transcriptase with a prime editing guide RNA (pegRNA) (54), allowing targeted base substitutions, insertions, and deletions through a template-directed mechanism (55, 56). By avoiding DSBs, both technologies reduce the risk of genomic instability while improving editing fidelity. Importantly, such approaches are particularly suited for correcting single-nucleotide variants and point mutations, which constitute a significant proportion of oncogenic alterations.

### 3.3 Modulation of tumor-supportive pathways

Beyond targeting immune cells or oncogenic drivers directly, CRISPR/Cas9 has also been applied to disrupt tumor-supportive pathways, particularly those that are associated with angiogenesis, extracellular matrix (ECM) remodeling, and metastatic progression (57). This strategy focuses on targeting the TME as an integrated system, rather than focusing solely on oncogenic drivers. One major area of intervention involves the inhibition of angiogenic signaling pathways, especially those mediated by vascular endothelial growth factor (VEGF) (58, 59). Disruption of VEGF-related pathways compromises tumor vascularization, leading to hypoxia, nutrient deprivation, and impaired tumor growth. Importantly, this approach highlights the dependency of tumors on sustained vascular support, positioning angiogenesis as a critical vulnerability that can be exploited through gene editing (60).

In parallel, CRISPR-mediated targeting of matrix metalloproteinases (MMPs) has demonstrated significant potential in inhibiting tumor invasion and metastasis through disruption of ECM remodeling (61). Functional studies have shown that CRISPR-mediated knockout of *MMP9* in cutaneous squamous cell carcinoma leads to reduced cellular viability and migratory capacity, accompanied by downregulation of multiple oncogenic signaling mediators, including *TGF-β, FGF, PI3K, VEGF-A*, and vimentin (62). Similarly, combined CRISPR editing of *MMP9* and miRNA-21 in prostate cancer models results in decreased tumor proliferation and invasion, alongside enhanced apoptotic regulation via BAX and mTOR pathways (63). These findings collectively illustrate that MMP disruption extends beyond structural ECM modulation to influence broader oncogenic signaling networks.

Notably, emerging evidence also suggests that MMPs may exhibit non-proteolytic functions that further contribute to tumor progression. For instance, CRISPR-based studies on *MMP3* have demonstrated that extracellular vesicles enriched with “moonlighting” MMP proteins can translocate into recipient cell nuclei and transactivate genes such as CCN2/CTGF, thereby promoting tumor-supportive transcriptional programs (64). This expands the functional landscape of MMPs beyond matrix degradation, positioning them as multifaceted regulators of tumor biology. Consequently, CRISPR-mediated disruption of MMPs not only impairs ECM degradation but also interferes with oncogenic signaling, epithelial–mesenchymal transition (EMT), and vascular remodelling, collectively reducing tumor aggressiveness and metastatic potential (61).

In addition to targeting proteolytic pathways, CRISPR-based strategies have been extended to the post-transcriptional regulatory level, particularly through the restoration of tumor-suppressive microRNAs. The reactivation of the let-7 family, for example, via CRISPR-mediated correction of mutations in miRNA precursors, enables simultaneous suppression of multiple oncogenic targets, including *HMGA2*, *RAS*, and *MYC* (65). This multi-target regulatory effect not only inhibits tumor proliferation but also suppresses EMT and metastatic progression. Importantly, the restoration of let-7 may indirectly modulate upstream regulators of MMP activity and EMT-associated pathways, thereby reinforcing its anti-metastatic function (66, 67).

## 4 Translational challenges and limitations of CRISPR/Cas9 in oncology

### 4.1 Safety considerations: off-target editing and immunogenicity

Although CRISPR/Cas9 demonstrates high editing efficiency *in vitro*, its clinical application remains challenging. These stem largely from the complexity of human physiological systems, including immune surveillance mechanisms, genomic instability, and the TME.

A key concern in CRISPR-based cancer therapies is the potential immunogenicity of the Cas9 protein. The RNA-guided Cas9 endonuclease is commonly derived from *Staphylococcus aureus* and *Streptococcus pyogenes*, raising concerns regarding its potential to trigger immunogenicity in the host body (4, 68). Findings from previous studies revealed that anti-Cas9 IgG antibodies against *S. aureus* Cas9 (SaCas9) and *S. pyogenes* Cas9 (SpCas9) were detected in healthy human samples (69, 70). This indicates that the human immune system has been exposed to Cas9 protein during bacterial infections, despite its nature as an intracellular protein. Consequently, this triggers the activation of both CD4+ and CD8+ cytotoxic T lymphocytes (CTLs) within the circulatory system, leading to the secretion of several key pro-inflammatory cytokines, including interferon-γ (IFN-γ), tumor necrosis factor-α (TNF-α), interleukin 2 (IL-2), and CD154. This immune response poses a potential risk of immune-mediated clearance of Cas9-expressing cells following CRISPR/Cas9 therapy in the human body, thereby deteriorating the efficacy of the treatment (70). Furthermore, the Cas9-induced CD4+ and CD8+ T-cell infiltration was found to be associated with the increased expression of muscle cytokine and a reduction of dystrophin gene expression in canine models. These lead to the elevation of muscle inflammation and cell death, which often result in conditions such as Duchenne muscular dystrophy (DMD) (71).

In addition, the specificity of CRISPR/Cas9 in the large mammalian genome remains a major hurdle, raising concerns about the off-target editing. A common cause of such off-target cleavage is the presence of base-pair mismatches, typically involving 3 to 5 bases between the protospacer adjacent motif (PAM)-distal part of the guide RNA (sgRNA) and target DNA (5, 72). Variations in the PAM sequence and Cas9 protein itself can significantly influence different propensities for off-target activities (12, 73). Interestingly, off-target cleavages could also happen under more complex mismatch conditions, especially near the ends of the target site, including: (i) the presence of 1 to 3 base mismatches with single-base bulge, (ii) DNA strands containing single-nucleotide bulges, and (iii) sgRNA harboring bulges of up to 4 nucleotides, facilitating cleavage despite imperfect base-pairing (6). These events may disrupt gene regulatory processes, thus activate proto-oncogenes, or result in chromosomal rearrangement, which contributes to poor safety and therapeutic outcomes.

Even when ideal targetability is achieved, the therapeutic outcome of the CRISPR/Cas9 system still remains a concern. Cancers are typically driven by complex interactions involving multiple genes and pathways. Henceforth, the knockdown of a single gene via CRISPR/Cas9 may not suffice in these cases and could even result in compensatory changes in the tumor or surrounding tissues (74). Additionally, redundancy in oncogenic pathways or the existence of feedback loops may render CRISPR-mediated edits ineffective or even deleterious (7).

### 4.2 Technical challenges in delivery to solid tumors

While the CRISPR/Cas9 genome-editing tool has shown great promise in cancer research and therapy, its application in solid tumors is significantly more complex and substantial further development is required. To date, there are several crucial technical challenges for the delivery of CRISPR/Cas9 system need to be addressed comprehensively.

Firstly, solid tumors are known by extensive genetic, epigenetic, and phenotypic heterogeneity, both between and within tumors. This intra-tumoral diversity complicates the design of universal guide RNAs, as single-target strategies may not address all relevant oncogenic drivers or mutated alleles within the same tumor (75). Compounding this, tumor plasticity which is often driven by stress-induced epigenetic reprogramming, may favor the dynamic alteration in oncogene expression in response to treatment or environmental changes. As a result, gRNA efficacy may be deteriorated, leading to reduced editing efficiency and potential resistance development (76).

Beyond static genetic diversity, tumor heterogeneity is a dynamic process driven by clonal evolution and adaptive selection pressures. Within a single tumor, genetically distinct subclonal populations frequently co-exist, each arising from different combinations of driver mutations, passenger alterations, epigenetic states, and therapeutic vulnerabilities (77). Under treatment stress, treatment-sensitive cells population may be eliminated while treatment-resistant subpopulations continue to expand with minimal impact from treatment, resulting in progressive clonal evolution and therapeutic resistance (78). This presents a unique challenge for CRISPR/Cas9-based interventions, as guide RNAs (gRNAs) designed against a single oncogenic target may only modify a subgroup of tumor cells, leaving genetically distinct clones unaffected (79, 80). Consequently, even highly efficient editing may not achieve durable tumor control if untreated subclones retain proliferative or metastatic potential. In addition, ongoing genomic instability may generate new mutations during tumor progression, further altering target sequence accessibility and potentially reducing gRNA binding efficiency over time.

The implications of tumor heterogeneity extend beyond target selection to the design of CRISPR-based therapeutic strategies. In many solid tumors, oncogenic signaling is maintained through parallel or redundant pathways, allowing cancer cells to compensate when a single target is disrupted (1). This biological redundancy may partially explain why monogenic targeting approaches often demonstrate limited efficacy despite successful editing. To address this challenge, increasing attention has been directed toward multiplex genome editing, whereby multiple oncogenic drivers, immune evasion pathways, or resistance-associated genes are simultaneously targeted using several gRNAs (8). Such approaches may reduce the likelihood of clonal escape by disrupting multiple adaptive routes available to tumor cells. This concept is already reflected in current clinical development programs, where multiplex editing strategies simultaneously target *PDCD1, TRAC, B2M, TGFBR2*, Regnase-1, *CISH*, or *HPK1* to enhance therapeutic durability and overcome mechanisms of adaptive resistance (**Table 1**). Nevertheless, increasing the number of editing targets also introduces additional complexity, including higher risks of chromosomal rearrangements, variable editing efficiencies across loci, and more demanding manufacturing and quality-control requirements (81, 82). Therefore, future CRISPR strategies will likely require integration with genomic profiling, single-cell sequencing, and longitudinal molecular monitoring to ensure that target selection accurately reflects the evolving clonal architecture of individual tumors (10).

Apart from concerns on the solid tumors, the TME plays a crucial impediment, where the presence of immunosuppressive cells, acidic pH, as well as stress-signaling, including hypoxia and inflammation, may limit the vector penetration, thus hindering the gene expression upon delivery of CRISPR components (10). Furthermore, oxidative stress and hypoxia-inducible signaling can downregulate promoters expressing Cas9 or gRNA, thereby reducing functional gene-editing activity even if the delivery is successful (10). In addition, the physical and physiological barriers, including dense extracellular matrices, irregular vasculature, and high interstitial fluid, may limit nanoparticle diffusion, uptake, and deep tissue infiltration. The presence of nuclease and protease in the blood circulatory system can rapidly degrade CRISPR components before reaching target sites, especially RNA and Cas9 protein (83). This urges the use of robust delivery vectors that are capable of shielding the loads en-route to tumor.

Additionally, the choices of delivery modalities of the present times pose challenges to the effective delivery with minimal off-target issues. The most direct method, which is electroporation, offers high transfection efficiency *in vitro*, but it is impractical for solid tumors due to its invasiveness, lack of targeting precision, and associated damage. Traditional viral vectors such as adeno-associated virus (AAV) face issues like immunogenicity and inefficacy due to low propensity for integration into the host genome. Moreover, their limited packaging capacity (~4.7kb) restricts the inclusion of Cas9 and regulatory elements in a single vector (83). Lentiviral vector is known for the risk of insertional mutagenesis, leading to concerns about safety and clinical applications (84, 85). Meanwhile, non-viral techniques such as nanoparticles showed advantages of relatively low immunogenicity and improved safety compared to viral vectors, but possess a certain level of challenges to be addressed (86). Lipid nanoparticles, which are known for its promising nucleic acid delivery, remain suboptimal due to limited tissue penetration and low intravascular stability. This results in rapid clearance from the blood circulation, indicating low delivery efficacy (87). Cationic polymeric-based or lipid-based vectors, which are frequently used for condensation of Cas9, may show a tendency to bind to negatively charged blood serum and form large aggregates, leading to rapid blood clearance and decreased biodistribution (83). In contrast to the above choices, exosomes and extracellular vesicles represent a rather ideal option with good biocompatibility, longer half-life in the circulatory system, and the capacity to penetrate through barrier layers. However, issues such as large-scale production and consistent cargo loading remain unresolved and present significant translational bottlenecks (88, 89). Overall, the application of most of the non-viral vectors is limited to *in vitro*, *ex vivo*, or local *in vivo* administration, as the efficiency of systemic administration of CRISPR/Cas9 remains debatable (83).

### 4.3 Ethical and regulatory considerations

CRISPR/Cas9 has been the most recent trending method for genome editing in various therapeutic fields, particularly cancer treatment. While genome editing offers unprecedented opportunities for precision oncology, its clinical implementation requires stringent assessment of patient safety, informed consent, long-term monitoring, and equitable access (90). These issues should be carefully evaluated because genome editing may lead to permanent biological changes, the consequences of which may not become fully apparent until years after treatment.

A fundamental ethical distinction in cancer applications is the difference between somatic and germline genome editing. Current CRISPR/Cas9 trials are exclusively limited to somatic cell editing, where genetic modifications are restricted to the treated patient and are not inherited by future generations (91). Nevertheless, significant concerns remain regarding the specificity and selectivity of CRISPR/Cas9 technology in editing hereditary cancer-associated genes in human germlines, such as mutated *BRCA1*, *BRCA2*, *MLH1*, and *CDH1*. Non-target mutations, chromosomal rearrangements, and long-term genomic stability, particularly as increasingly complex multiplex editing strategies could possibly lead to detrimental genetic drift in future generations (92). The efficacy of this technology can be achieved through various strategies, including *in silico* prediction, implementation of alternative delivery methods, modifying gRNAs, selecting an eligible CRISPR nuclease, and reassessment of the suitability of target sites (11). However, the safety and potential risks associated with these improvised techniques in clinical and therapeutic applications remain debatable.

Another critical bioethical issue of the application of CRISPR/Cas9 treatment in germlines is genetic mosaicism as a possible side effect of inefficient cutting of the target gene, or cell division initiates before the completion of genome editing (93). Notable examples to be highlighted include mosaicism observed in *G6PD* and *MYBPC3^ΔGAGT^* in human embryos, as well as the endogenous β-globin gene (*HBB*) in tripronuclear (3PN) zygotes (94–96). Additionally, the potential misuse of CRISPR/Cas9 for non-therapeutic purposes may lead to genetically improved populations or individuals in developed countries, resulting in biased benefits in comparison to others in terms of intelligence and physical features (97).

In addition, informed consent represents a particularly complex ethical consideration in CRISPR/Cas9-based cancer therapies. Unlike conventional pharmacological interventions, genome editing approaches may induce permanent genetic alterations which long-term biological consequences remain incompletely understood. Consequently, consent processes should extend beyond discussion of anticipated therapeutic benefits to include uncertainties surrounding off-target editing, genomic instability, clonal expansion, secondary malignancies, durability of edited-cell persistence, and the potential need for prolonged post-treatment surveillance (98, 99). Given that definitive safety assessments often require long-term follow-up, patients enrolled in early-phase clinical trials must also be informed that therapeutic benefit may be limited and that participation primarily contributes to evaluating feasibility and safety rather than clinical efficacy (99). These considerations highlight the importance of transparent risk communication, ongoing patient engagement, and robust regulatory oversight to ensure responsible clinical translation of genome-editing technologies.

Long-term surveillance has therefore emerged as a critical component of responsible clinical translation reflecting the recognition that the full spectrum of genome editing outcomes may not be apparent during initial clinical evaluation. Regulatory authorities, including the U.S. Food and Drug Administration (FDA) and the European Medicines Agency (EMA), have therefore recommended prolonged follow-up programs to establish comprehensive safety profiles and generate real-world evidence on the durability and biological consequences of genome-editing therapies (99). Such surveillance not only safeguards patient welfare but also provides critical data to refine future therapeutic strategies and risk-assessment models.

In parallel, regulatory oversight of CRISPR-based cancer trials continues to evolve alongside technological innovation. Current frameworks require comprehensive evaluation of editing efficiency, off-target activity, manufacturing consistency, vector safety, and product characterization before clinical approval (97). However, emerging technologies such as base editing, prime editing, and *in vivo* genome editing may introduce new regulatory considerations that are not fully addressed by existing guidelines. As genome-editing technologies become increasingly sophisticated, regulatory frameworks must remain adaptive, integrating advances in genomic monitoring, computational risk prediction, and post-marketing surveillance. Sustained collaboration among scientists, clinicians, regulators, patients, and bioethicists will therefore be essential to develop evidence-based governance models that balance innovation with patient safety and public trust.

Finally, equity and accessibility will become increasingly important as CRISPR-based therapies move toward clinical implementation. Current approaches, particularly autologous cell therapies, require complex manufacturing processes and substantial financial investment, potentially limiting access across healthcare systems and regions (100). Beyond cost, disparities in genomic testing, specialized treatment centers, and clinical trial participation may further exacerbate inequities in cancer care. Therefore, ethical evaluation of CRISPR-based oncology should consider not only safety and efficacy but also equitable access. Advances in scalable manufacturing, allogeneic platforms, and standardized delivery technologies will be essential to improve both clinical feasibility and broader patient accessibility (90). In short, these challenges highlight the multifactorial barriers that continue to influence the clinical translation of CRISPR/Cas9-based cancer therapies. While considerable progress has been made in improving editing precision, delivery efficiency, and safety, several technical, biological, manufacturing, and regulatory hurdles remain unresolved. A summary of the major challenges and the current strategies being investigated to address them is shown in **Table 2**.

**Table 2:** Major challenges affecting the clinical translation of CRISPR/Cas9-based cancer therapies and current mitigation strategies.

Challenge	Impact on clinical translation	Current mitigation strategies	References
Off-target editing	Unintended genomic alterations and safety concerns	High-fidelity Cas variants, optimized gRNA design, alternative editing platforms	(5, 6)
Delivery limitations	Reduced editing efficiency, particularly in solid tumors	Viral vectors, lipid nanoparticles, tumor-targeted nanocarriers, engineered exosomes	(26, 41, 85)
Tumor heterogeneity and clonal evolution	Incomplete tumor targeting and therapeutic escape	Multiplex editing, patient-specific target selection, single-cell profiling	(8, 77, 101)
Adaptive resistance	Activation of compensatory oncogenic pathways	Combination therapies and multi-target editing strategies	(32, 78, 102)
Immunogenicity	Immune responses against Cas proteins or delivery vehicles	*Ex vivo* editing approaches, transient delivery systems	(29, 40, 42)
Variable editing efficiency and genomic instability	Inconsistent therapeutic outcomes and safety concerns	Base and prime editing, optimized editing platforms	(54, 103)
Manufacturing scalability	Increased production complexity and treatment costs	Allogeneic cell platforms, standardized manufacturing workflows	(16, 100)
Regulatory and ethical considerations	Challenges in clinical implementation and approval	Long-term monitoring, regulatory oversight, quality-control frameworks	(91, 104)

## 5 Future directions and translational opportunities

### 5.1 Precision editing technologies

The continued evolution of CRISPR-based technologies in oncology reflects a transition from proof-of-concept applications toward more precisely engineered and clinical-ready strategies. Rather than representing a single therapeutic modality, CRISPR is now known as a flexible gene-editing tool, which is capable of interfacing with multiple layers of cancer biology. This transition is driven not just by the advances in editing chemistry, but also by a growing recognition that effective cancer therapies require coordinated modulation of complex and interconnected pathway networks rather than on targeting individual genes (75).

A major area of CRISPR-based innovation lies in the development of next-generation editing platforms, particularly base editing and prime editing, which aim to address key limitations associated with DSB in conventional CRISPR/Cas9 systems. By enabling targeted nucleotide conversions without inducing DNA breaks, base editors show improved editing precision with reduced risk of unintended genomic modifications. Prime editing further expands this capability by enabling a broader spectrum of genetic modifications, including all possible base substitutions as well as small insertions and deletions (103). Importantly, these technologies reflect a progression from gene disruption toward fine-tuned genomic sequence modification, which may be more compatible with the heterogeneous nature of cancer-associated genomic alterations. However, their therapeutic applicability remains contingent upon improvements in delivery efficiency and editing consistency across diverse tumor contexts (10).

Beyond base and prime editing, the CRISPR technology is expanding beyond conventional Cas9-mediated DNA editing to include alternative CRISPR effectors with functionalities. Cas12 nucleases offer distinct PAM requirements and cleavage characteristics that may improve targeting flexibility, while Cas13 systems enable programmable RNA editing without permanent genomic modification (105, 106). These RNA-targeting approaches are particularly attractive in oncology because they allow transient modulation of oncogenic transcripts and may reduce concerns regarding irreversible genomic alterations. In parallel, CRISPR-based epigenome editing platforms, which employ catalytically inactive Cas proteins fused to transcriptional or epigenetic regulators, are emerging as tools to modulate gene expression without altering the underlying DNA sequence (107). Such approaches may be particularly valuable in cancers driven by epigenetic dysregulation, where therapeutic benefit may be achieved through reversible reprogramming of transcriptional states rather than permanent genome modification (107).

### 5.2 Complementary therapeutic technologies

Beyond advances in editing strategy, the future impact of CRISPR in oncology will likely depend on its integration within combinatorial treatments. In the context of immunotherapy, CRISPR has already demonstrated substantial contributions in enhancing the efficacy of adoptive cell therapies through precise and multiplexed genetic modifications (40, 41). However, future developments are likely to extend toward more sophisticated engineering strategies that simultaneously address antigen recognition, immune persistence, and resistance to tumor-induced suppression. While most clinical studies have relied on *ex vivo* editing approaches due to their controllability and established manufacturing workflows, direct *in vivo* delivery could substantially expand the applicability of genome editing, particularly for solid tumors where cellular therapies face significant logistical and biological constraints. For example, viral vectors such as adeno-associated viruses (AAV) vectors continue to play a role in applications requiring high transduction efficiency, although concerns related to payload limitations and immunogenicity persist (84, 85). Additionally, a particularly important area of development is the advancement of *in vivo* delivery platforms, which aim to overcome one of the principal limitations of current CRISPR-based cancer therapies. Among the most promising non-viral systems are lipid nanoparticles (LNPs), which can encapsulate Cas9 mRNA, guide RNAs, or ribonucleoprotein complexes and facilitate intracellular delivery while reducing the risk of insertional mutagenesis associated with viral vectors. The successful application of LNP-mediated CRISPR delivery in transthyretin amyloidosis has provided proof-of-principle that systemic genome editing can be achieved in humans, thereby accelerating efforts to adapt similar platforms for oncology applications (108). Supporting this translational potential, preclinical studies have demonstrated the feasibility of LNP-mediated CRISPR delivery in cancer models.

Beyond conventional LNPs, tumor-targeted nanocarriers represent an increasingly important area of innovation. These systems incorporate targeting ligands, antibodies, peptides, or aptamers to enhance preferential accumulation within tumor tissues while minimizing exposure to healthy organs. For example, a recently developed micelleplex system demonstrated efficient delivery of CRISPR/Cas9 components targeting mutant *KRAS* in lung cancer models, resulting in significant gene editing and tumor growth suppression. Such studies illustrate how advances in nanocarrier design are beginning to address long-standing challenges related to tumor-specific delivery, thereby strengthening the translational potential of *in vivo* CRISPR-based oncology therapies (109).

Another promising delivery modality involves extracellular vesicles and engineered exosomes, which offer several biological advantages over synthetic nanoparticles. Exosomes possess intrinsic biocompatibility, low immunogenicity, and natural intercellular transport capabilities, making them attractive vehicles for CRISPR delivery. Recent studies have demonstrated successful packaging of Cas9 ribonucleoprotein complexes into engineered exosomes capable of inducing targeted gene disruption (e.g: *KrasG12D*, and *KAT5*) in tumor cells both *in vitro* and *in vivo* (110, 111). Furthermore, chondrocyte affinity peptide (Cap)-modified exosomes derived from mesenchymal stem cells (MSCs) were successfully used to deliver CRISPR/Cas9 system for Asporin (ASPN) knockout in osteoarthritis models (112). While these studies remain largely pre-clinical, and may differ substantially from cancer, this study highlights the feasibility of exosome-mediated CRISPR delivery and supports ongoing efforts to adapt engineered extracellular vesicles for tumor-targeted genome editing applications.

In parallel, emerging CRISPR platforms are increasingly incorporating base editors and prime editors into *in vivo* delivery strategies. Because these technologies avoid double-strand DNA breaks, they may offer improved safety profiles for direct administration in patients. Preclinical studies have demonstrated the ability of base editors to precisely target oncogenic mutations, specifically *KRAS* and *TP53*, and immune regulatory pathways using both viral and non-viral delivery systems (113, 114). While oncology-specific applications remain at an early stage, these developments suggest that future *in vivo* cancer therapies may extend beyond gene disruption toward precise correction of pathogenic mutations and transcriptional reprogramming.

The future development of CRISPR-based cancer treatments is also likely to be accelerated by advances in computational biology and functional genomics. Artificial intelligence (AI)-assisted guide RNA design platforms are increasingly being used to optimize target selection, predict off-target activity, and improve editing efficiency (115). By integrating large genomic datasets with machine-learning algorithms, these approaches may facilitate the development of more precise and patient-specific editing strategies. Similarly, single-cell CRISPR screening technologies now enable simultaneous interrogation of gene function and cellular phenotypes at single-cell resolution, providing unprecedented insight into tumor heterogeneity, mechanisms of immune evasion, and therapeutic resistance (101). These platforms are increasingly being used to identify novel vulnerabilities that may subsequently be translated into CRISPR-based therapeutic targets.

An emerging area of focus is the integration of CRISPR technologies with synthetic biology to develop programmable therapeutic systems capable of detecting, processing, and responding to complex tumor-associated signals. CRISPR-based synthetic gene circuits can be designed to function as biological logic gates, permitting therapeutic responses only when specific combinations of cancer biomarkers are identified (116, 117). These programmable systems may improve tumor targeting, reduce off-target editing, and enhance the safety profile of gene-editing treatments (118, 119). In immuno-oncology, these concepts are increasingly influencing the design of next-generation cellular therapies. Although current clinical-stage CRISPR therapies remain focused on multiplex genome editing rather than fully synthetic gene circuits, several engineered cell products already incorporate multiple coordinated genetic modifications to improve persistence, reduce exhaustion, and enhance anti-tumor activity. For example, next-generation allogeneic CAR-T candidates such as CTX112 and CTX131 employ multiplex editing strategies that extend beyond conventional CAR insertion to optimize cellular fitness and therapeutic durability (32). Looking ahead, emerging preclinical platforms incorporating logic-gated receptors, inducible effector functions, and TME-responsive regulatory circuits may further advance the precision and safety of CRISPR-based immunotherapies, enabling context-dependent therapeutic responses that are currently not achievable with conventional cellular engineering approaches (120, 121).

Beyond its application as a standalone therapeutic platform, CRISPR is increasingly being explored as a means of enhancing the efficacy of existing cancer treatments. In immuno-oncology, genome editing may complement immune checkpoint blockade by improving T-cell persistence, overcoming tumor-induced immunosuppression, and restoring anti-tumor activity in resistant tumors. Similarly, CRISPR-mediated disruption of resistance-associated genes and signaling pathways has shown potential to sensitize tumor cells to radiotherapy, targeted therapies, and other conventional treatment strategies (102). This reflects a broader trend toward integrating genome editing within multimodal treatment frameworks, where CRISPR functions not only as a direct therapeutic intervention but also as a strategy to overcome adaptive resistance and improve treatment responsiveness. Such combinatorial approaches may be particularly valuable in aggressive and heterogeneous cancers, where single-agent therapies often provide limited or transient clinical benefit.

### 5.3 Personalized and scalable CRISPR-based cancer treatments

The increasing recognition of tumor heterogeneity has reinforced the need for personalized therapeutic strategies capable of addressing patient-specific molecular abnormalities. In this regard, CRISPR offers a level of programmability that is difficult to achieve with conventional therapies, enabling the design of interventions tailored to distinct genetic mutational profiles and resistance mechanisms. However, the implementation of such personalized approaches is closely linked to challenges in scalability, manufacturing complexity, and cost (122). Current cell-based therapies, including CAR-T cell therapies, remain resource-intensive as they require extensive *ex vivo* manipulation and are often constrained by variability in patient-derived cells (123). As such, future innovations are likely to focus on the development of standardized and scalable platforms, including allogeneic cell products and *in vivo* editing strategies, which could broaden accessibility while maintaining therapeutic specificity.

## 6 Conclusion

CRISPR/Cas9 has rapidly transitioned from a foundational genome-editing tool to a versatile platform with increasing relevance in cancer research and therapy. As highlighted throughout this review, its application in oncology is characterized by combining various strategies that are mechanistically diverse yet functionally complementary, including immune cell engineering, direct modulation of oncogenic alterations, and disruption of tumor-supportive pathways. To date, the most encouraging clinical outcomes have been observed in hematological malignancies, particularly B-cell malignancies and multiple myeloma, where *ex vivo* CRISPR-edited cellular therapies have demonstrated favorable safety profiles, sustained persistence of edited cells, and early evidence of antitumor activity. In contrast, clinical translation in solid tumors remains comparatively limited despite promising preclinical findings, reflecting persistent challenges associated with tumor-selective delivery, intratumoral heterogeneity, and the immunosuppressive TME. Nevertheless, expanding efforts in solid tumors and virus-associated cancers also reflect the gradual expansion of therapeutic scope of CRISPR-based interventions beyond hematological settings.

Despite encouraging clinical progress, several critical barriers continue to limit the widespread adoption of CRISPR-based cancer therapies. Among these, efficient and tumor-selective delivery remains arguably the most significant challenge, particularly for solid tumors where physical barriers, heterogeneous target expression, and the TME restrict editing efficiency. In parallel, concerns regarding off-target editing and long-term genomic stability continue to influence both safety assessment and regulatory decision-making. Manufacturing scalability also remains a major consideration, especially for autologous cell-based products that require complex and resource-intensive production workflows. Furthermore, the regulatory guidelines for genome editing continues to evolve, with increasingly stringent evaluation surrounding product characterization, long-term follow-up, and quality control requirements (31). Overall, these challenges highlight that successful clinical translation will depend not only on advances in editing precision but also on the development of scalable manufacturing platforms, robust delivery technologies, and regulatory frameworks capable of supporting emerging editing modalities.

Looking forward, the future impact of CRISPR/Cas9 in oncology will likely depend on its integration with emerging technologies, including advanced delivery systems and existing combinatorial therapeutic strategies. Ultimately, the success of CRISPR/Cas9 in oncology will depend on its ability to balance precision with scalability and safety concerns, thereby establishing its role as a core component of next-generation cancer therapeutics.
